# First identification of *Mycobacterium avium* subsp. *paratuberculosis* in wild ruminants in a zoo in Mexico

**DOI:** 10.14202/vetworld.2022.655-661

**Published:** 2022-03-23

**Authors:** A. L. Hernández-Reyes, G. Chávez-Gris, E. Maldonado-Castro, L. E. Alcaraz-Sosa, M. T. Díaz-Negrete

**Affiliations:** 1 National Autonomous University of Mexico (UNAM), School of Veterinary Medicine and Zootechnics (FMVZ), Center for Teaching, Research and Extension of Animal Production in High Plateau (CEIEPAA), Tequisquiapan-Ezequiel Montes Highway Km 8.5, 76790 Tequisquiapan, Queretaro, Mexico; 2 Autonomous Metropolitan University, Xochimilco Campus, Division of Biological and Health Sciences, Department of Agricultural and Animal Production, Veterinary Medicine and Zootechnics, Calzada del Hueso 1100, Col, Villa Quietud, Coyoacán, 09460, Mexico City, Mexico; 3 Zoological and Wildlife Conservation General Directorate (DGZCFS), Secretariat of Environment of Mexico City, Mexico City, Mexico

**Keywords:** histopathology, IS*900*, isolation, paratuberculosis, *scimitar-horned oryx*, zoo animals

## Abstract

**Background and Aim:**

Paratuberculosis (PTB) is an infectious disease that induces chronic enteritis in ruminants. It is caused by *Mycobacterium avium* subsp. *paratuberculosis* (MAP). In this study, we evaluated the presence of MAP using bacteriological, molecular, and anatomopathological studies, based on the clinical suspicion of PTB in a zoo, in an area housing 10 scimitar-horned oryx (*Oryx dammah*), five giraffes (*Giraffa camelopardalis*), and three blue wildebeests (*Connochaetes taurinus*).

**Materials and Methods:**

From November 2016 to June 2017, fecal samples were collected from individuals of the three species on four occasions, resulting in a total of 56 fecal samples. In addition, five small intestine samples were collected from the necropsies of three adult scimitar-horned oryx females and two oryx calves. MAP identification was performed through isolation in Herrold’s medium with egg yolk, mycobactin, and sodium pyruvate, Ziehl–Neelsen staining, IS*900* polymerase chain reaction (IS*900* PCR), and anatomopathological examination of intestine samples.

**Results:**

Diffuse granulomatous enteritis with abundant acid-fast bacilli was found in two out of five intestine samples from adult scimitar-horned oryx females. MAP was isolated in 7/56 (12.5%) of the fecal samples from four scimitar-horned oryx, one giraffe, and two wildebeest samples. Two out of 5 (40%) samples obtained from scimitar-horned oryx tested positive. IS*900* PCR yielded five positive samples (two fecal samples and three small intestine samples). MAP isolates were classified as Type C (Cattle) using type-specific PCR.

**Conclusion:**

These results demonstrated the presence of MAP in the area evaluated and indicated the importance of both sampling live animals and conducting postmortem examinations. The use of bacteriological and histopathological diagnostic techniques demonstrated in this study will provide insight into the health status and prevalence of paratuberculosis in wild ruminants under human care.

## Introduction

Paratuberculosis (PTB) is an infectious disease that induces chronic enteritis. It is caused by *Mycobacterium avium* subsp. *paratuberculosis* (MAP), an acid-fast bacilli (AFB) [1,2] affecting both domestic and wild ruminants [3,4], which causes negative effects on animal health and welfare and produces significant economic losses, mainly in the dairy industry [5]. The primary transmission pathway is fecal-oral, although other paths have been described, such as vertical transmission [6,7]. MAP has an extended incubation period that can last anywhere between 2 and 7 years. After the incubation period, it causes progressive weight loss, intermandibular edema, diarrhea or viscous feces, as well as granulomatous lesions mainly in the jejunum, ileum, and ileocecal valve [8]. Infected animals release MAP intermittently through their feces, starting from the initial infection phases, even if the animals do not show signs of illness. This shedding of bacteria makes it challenging to control the disease. The identification of MAP requires the use of one or more diagnostic tests. Bacterial cultures, polymerase chain reaction (PCR), and antibody detection ELISA tests are among the test most widely used to identify MAP [9,10].

MAP has been reported in several wild species, including ruminants, carnivores, lagomorphs, and birds. Wild ruminants predominantly show clinical signs such as diarrhea and weight loss, similar to that reported in domestic ruminants [4,11]. The studies on wild ungulates affected with PTB under human care have found that conditions such as high animal density and limited enclosure areas may facilitate exposure to various infectious agents, including MAP [12,13]. PTB could have a negative effect on conservation programs [14], since it has been detected in species with high genetic value, such as the African south-central black rhinoceros (*Diceros bicornis minor*) [15] and the scimitar-horned oryx (*Oryx dammah*), the latter of which is extinct in the wild [16,17].

Two scimitar-horned oryx from a zoo of Mexico were suspected for PTB based on clinical symptoms. Hence, this study aimed to detect the presence of MAP using bacteriological and molecular techniques, together with anatomopathological studies in three species of ungulates that share an enclosure in a zoo in Mexico, scimitar-horned oryx, giraffes (*Giraffa camelopardalis*) and blue wildebeests (*Connochaetes taurinus*).

## Materials and Methods

### Ethical approval

Ethical approval was not required for this study; however, fecal samples were collected as per standard sample collection procedure without any harm to animals.

### Study period and location

This study was conducted from November 2016 to June 2017. The fecal samples were obtained from a zoo in Mexico City; subsequently, the samples were processed at a Research Center at the Faculty of Veterinary Medicine at National Autonomous University of Mexico.

### Animal study subjects

In 2016, two scimitar-horned oryx females in a zoo in Mexico were reported to have diarrhea and weight loss, a clinical picture compatible with PTB. These two females inhabited an enclosure with another eight scimitar-horned oryx (six adults and two calves born during the study period), five giraffes, and three wildebeests. Each animal was identified based on its anatomical characteristics and assigned a letter with the initial of the species’ common name followed by a consecutive number for each individual: Oryx O1 through O10; giraffe G1 through G5; and wildebeest W1 through W3.

### Obtaining fecal samples

Fecal samples were obtained from all animals with a 2-month interval on four separate occasions: (1) November-December; (2) January-February; (3) March-April; and (4) May-June using a non-invasive method of collecting feces from the substrate immediately after defecation. Each sample was identified and stored at −20°C until used in bacterial cultures and IS*900* PCR tests.

### Anatomopathological studies

Necropsies were performed on five animals (three adults and two calves) belonging to the study population that died from November 2016 to June 2017. Sections of jejunum and ileum were collected and fixed in 10% buffered formalin (pH 7.6) with a posterior inclusion in paraffin, sectioned into 4 mm thick slices, and stained with hematoxylin and eosin and Ziehl–Neelsen (ZN) stains. Sections of the intestine showing apparent mucosal thickening were obtained during the necropsies and maintained at −20°C until their use in bacterial cultures and PCR tests.

### Cultures

Decontamination of fecal and intestinal samples was performed with 0.75% hexadecylpyridinium chloride before sample inoculation [18]. Usually, before culture MAP, a decontamination procedure has to be done to reduce other bacteria that can inhibit MAP survival. To optimize MAP identification, before decontamination, the mycobacterial concentration was measured from the intestinal mucosa samples, using the procedure described by Ratnamohan and Spencer [19], and modified by Estévez-Denaives *et al*. [20].

Fecal and intestine samples were inoculated into Löwenstein–Jensen (LJ; Difco, USA) medium and Herrold’s medium (Becton, Dickinson Bioxon, Mexico) with egg yolk, sodium pyruvate (Affymetrix USB products, USA), and myco­bactin J (Diag. Reag. VSLAPHIS, Ame, la, USA) at a concentration of 2 mg/L. The samples were also inoculated into LJ medium and HEYP without mycobactin. The test tubes were incubated (Incucell, Germany) at 37°C for 52 weeks [18,20].

### ZN stain in fecal smears and intestinal mucosa maceration smears

ZN stain (Carbol Fuchsin, Hycel, Mexico; Acid alcohol, JT Baker, USA; Methylene blue, Hycel, Mexico) smears were performed to detect AFB from fecal and intestinal mucosa samples [20].

### IS*900* PCR on fecal and intestinal mucosa samples

#### Feces

Cellular lysis and DNA extraction from fecal samples were performed in accordance with the procedure described by Garrido *et al*. [21]. Briefly, from fecal decontamination, 10 mL of the upper aqueous phase was transferred into 15 mL tubes and centrifuged (Thermo Scientific, Sorvall Legend Micro 21 centrifuge, Germany); after that, the pellet was washed in phosphate-buffered saline (PBS: pH 7.2) three times. The pellet was resuspended in 2 mL of PBS, after that was centrifuged at 9600 x g. The pellet was resuspended with TE-triton (Sigma, USA) 100X, followed by three freezing cycles (5 min in liquid nitrogen) and boiling [5 min at 100°C in a dry bath (Boekel Scientific, USA)]. After this, 450 mL of Guanidine isothiocyanate (Gibco BRL, USA) and 250 mL of Ammonium acetate (JT Baker, USA) 7.5 M, pH 6.3 were added and mixed by inversion. The following steps were repeated twice: addition of 500 mL of Chloroform (JT Baker) Isoamyl alcohol (JT Baker) (24:1), mix by inversion, centrifugation at 9600 g, and transfer of the upper aqueous phase into a new tube. The DNA was precipitated with Isopropanol (JT Baker) and washed twice with 70% Ethanol (JT Baker). The pellet was air-dried and resuspended with 20-30 μL of water.

#### Intestine

Cellular lysis and DNA extraction from the intestine were performed using intestinal mucosa samples [20] with a commercially available extraction kit (*QIAamp DNA mini Kit spin column*, QIAGEN^®^, Dusseldorf, Germany).

### PCR

#### IS900 PCR

The P3N (5´GGGTGTGGCGTTTTCCTTCG 3´) and P5N (5´ATTTCGCCGCCACCGCCACG 3´) primers were used. These amplify a fragment of 314 bp from the IS*900* insertion sequence of MAP [22,23]. For this test, 3 μL of DNA were used (equivalent to 0.5 μg), 20 μL of FastStart™ PCR Master (Roche^®^, USA), and 1 μL of each primer. The PCR protocol was 94°C for 5 min, followed by 35 cycles at 94°C for 40 s, 56°C for 40 s, and 72°C for 40 s, with a final cycle at 72°C for 5 min.

#### Type-specific PCR

PCR was performed on positive IS*900* PCR samples using the DMC 529 (5′-GCTGTTGGCTGCGTCATGAAGTC-3′) and DMC 531 (5′-TTCTTATCGGACTTCTTCTGG-3′) primers that amplify a 310 bp product for Type C and a 162 bp for Type S [24]. This test used 3 μL of DNA, 20 μL of FastStart™ PCR Master (Roche), and 1 μL of each primer. The PCR conditions were 95°C (203°F) for 3 min, followed by 35 cycles at 94°C (201.2°F) for 30 s, 60°C (140°F) for 30 s, and 72°C (161.6°F) for 30 s, with a final cycle at 72°C (161.6°F) for 5 min.

## Results

### Anatomopathological studies

**Figure-1:**
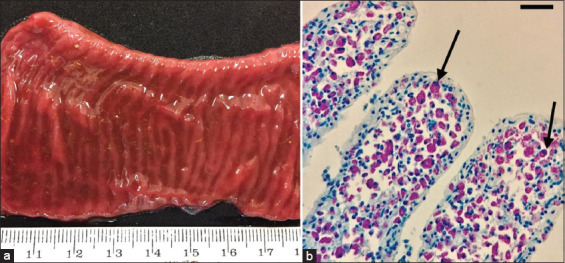
Scimitar-horned oryx (O6). (a) Small intestine section showing a dark red coloration and diffuse thickening of the intestinal mucosa. (b) Abundant macrophages with significant numbers of acid-fast bacilli inside (arrows) in the jejunal lamina propria. Ziehl–Neelsen, 40×. Scale bar 50 μm.

Necropsies were performed on five individuals: Three adult scimitar-horned oryx females (O2, O6, and O8) and two scimitar-horned oryx calves between 1 and 2 weeks of age (O9 and O10). The most relevant macroscopic findings were thickening and reddening the small intestine mucosa (Figure-1a) in two adult females (O6 and O8), which showed signs compatible with PTB. The O2 adult female showed advanced postmortem changes, and the two calves presented moderate congestion of the intestinal mucosa.

Under the compound light microscope (40x, Leica DM 1000, USA), the O6 and O8 females exhibited severe diffuse granulomatous enteritis in the jejunum and ileum, with abundant AFB inside the macrophages visualized using the ZN stain (Figure-1b). The other three animals (O2, O9, and O10) did not show any apparent histopathological changes.

### Culture

#### Fecal cultures

**Figure-2:**
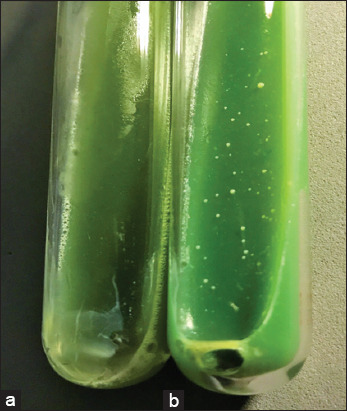
(a) Herrold’s medium with egg yolk and sodium pyruvate (HEYP) with no bacterial colony growth. (b) Herrold’s medium with egg yolk, sodium pyruvate, and mycobactin (HEYMP) showing colony growth compatible with *Mycobacterium avium* subsp. *paratuberculosis* at week 10, originating from scimitar-horned oryx fecal sample.

From a total of 56 fecal samples collected, 24 belonged to scimitar-horned oryx, 20 to giraffes, and 12 to wildebeests. A total of 7/56 (12.5%) bacteriological isolates were obtained from the samples processed: Four from scimitar-horned oryx, two from wildebeests, and one from giraffes (Table-1). Isolates were obtained from HEYMP between 8 and 10 weeks of incubation (Figure-2), except for three cases: One from a giraffe and two from wildebeests, in which isolates were obtained until 50 weeks of incubation in HEYMP medium. Four isolates were obtained from the first fecal sample collection period: One from oryx, one from giraffe, and two from wildebeests. The remaining three isolates were obtained from the fourth fecal sample collection period (Table-2). No MAP isolates were obtained from LJ growth media with mycobactin.

**Table-1:** MAP detection in feces and intestine using IS*900* PCR, Ziehl–Neelsen (ZN) stain, and bacterial cultures in scimitar-horned oryx (*Oryx dammah*), giraffe (*Giraffa camelopardalis*), and wildebeest (*Connochaetes taurinus*). (Grayed-out cells: No necropsy performed).

Species	Feces			Intestine		
	
	Culture % (+/total)	ZN % (+/total)	IS*900* PCR % (+/total)	Culture % (+/total)	ZN % (+/total)	IS*900* PCR % (+/total)
Scimitar-horned oryx	7.1% (4/56)	3.5% (2/56)	3.5% (2/56)	40% (2/5)	60% (3/5)	60% (3/5)
Giraffe	1.7% (1/56)	0	0			
Wildebeest	3.5% (2/56)	0	0			
Total	12.5% (7/56)	3.5% (2/56)	3.5% (2/56)	40% (2/5)	60% (3/5)	60% (3/5)

ZN=Ziehl–Neelsen, PCR=Polymerase chain reaction, MAP=*Mycobacterium avium* subsp. *paratuberculosis*

**Table-2:** ZN stain, IS*900* PCR, and bacterial culture results from feces and intestine samples in scimitar-horned oryx (*Oryx dammah*), giraffe (*Giraffa camelopardalis*), and wildebeest (*Connochaetes taurinus*)

Animal ID	Species	Sex	Feces			From necropsy (intestine)		
	
			IS*900* PCR	ZN stain	HEYMP culture	IS*900* PCR	ZN stain	HEYMP culture
O1	*Oryx dammah*	M	-	-	-	NO	NO	NO
O2 Nx	*Oryx dammah*	F	-	-	-	-	-	-
O3	*Oryx dammah*	M	-	+*^4^	+*^4^	NO	NO	NO
O4	*Oryx dammah*	M	-	-	+*^4^	NO	NO	NO
O5	*Oryx dammah*	F	-	-	-	NO	NO	NO
O6 Nx	*Oryx dammah*	F	+*^1^	+*^1^	+*^1^	+	+	+
O7	*Oryx dammah*	F	-	-	+*^4^	NO	NO	NO
O8 Nx	*Oryx dammah*	F	+*^1^	-	-	+	+	+
O9 (calf) (Nx)	*Oryx dammah*	NS	NO	NO	NO	+	+	-
O10 (calf) (Nx)	*Oryx dammah*	NS	NO	NO	NO	-	-	-
G1	*Giraffa camelopardalis*	M	-	-	-	NO	NO	NO
G2	*Giraffa camelopardalis*	F	-	-	-	NO	NO	NO
G3	*Giraffa camelopardalis*	M	-	-	+**^1^	NO	NO	NO
G4	*Giraffa camelopardalis*	F	-	-	-	NO	NO	NO
G5	*Giraffa camelopardalis*	F	-	-	-	NO	NO	NO
W1	*Connochaetes taurinus*	M	-	-	+**^1^	NO	NO	NO
W2	*Connochaetes taurinus*	F	-	-	+**^1^	NO	NO	NO
W3	*Connochaetes taurinus*	M	-	-	-	NO	NO	NO

+=Positive, -=Negative, ID=Identification, O=Scimitar-horned oryx , G=Giraffe, W=Wildebeest, F=Female, M=Male, NS=Not specified, Nx=Necropsy performed, NO=Not obtained.

* bacterial colony growth between 8 and 10 weeks

** bacterial colony growth at 50 weeks. The sample collection period from which growth was obtained is indicated with a superscript number in the table, 1: November-December, 2: January-February, 3: March-April, 4: May-June, ZN=Ziehl–Neelsen, PCR=Polymerase chain reaction

#### Intestinal sample cultures

MAP isolates were obtained from 2/5 (40%) of the intestine samples between incubation weeks 8 and 10 on HEYMP, from two adult females (O6 and O8) (Table-2). The isolates were confirmed using IS*900* PCR. No MAP isolates were obtained from LJ growth media with mycobactin.

#### ZN stain on fecal smears and intestinal mucosa macerations

Of the 56 fecal samples tested, only two adult oryxes (O6 and O3) presented with AFB (3.5%). The intestine samples resulted in three positives (60%) from two adult scimitar-horned oryx females (O6 and O8), and one calf of the same species (O9) (Table-2).

#### IS900 PCR detection from feces and intestine samples

**Figure-3:**
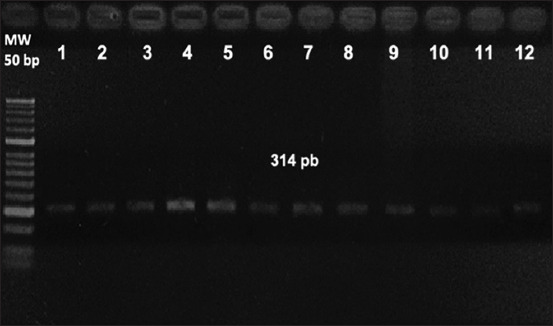
IS*900* polymerase chain reaction results. MW well; Molecular weight marker 50 bp; wells (1-6) amplification products (314 bp) obtained from DNA extraction of the bacterial colonies developed from fecal and intestinal mucosa samples; wells (7-9) amplification products (314 bp) obtained from DNA extraction of intestinal mucosa samples; wells (10-11) amplification products (314 bp) obtained from DNA extraction of fecal samples; and well (12) positive control of IS*900* fragment. All samples are from scimitar-horned oryx.

Amplification of the IS*900* sequence was achieved in 2/56 (3.5%) fecal samples belonging to two adult scimitar-horned oryx females (O6 and O8), obtained during the first sample collection period. In addition, amplification of the IS*900* sequence was achieved in 3/5 (60%) intestine samples, belonging to two adult scimitar-horned oryx females (O6 and O8) and one oryx calf (O9) (Table-2 and Figure-3).

#### Type-specific PCR

Type-specific PCR was performed from all nine bacterial culture isolates, seven from feces and two from intestine samples, resulting in the amplification of the 310 bp segment from Type C.

The products amplified from IS*900* PCR after isolation were sequenced and compared against the BLAST database [25], producing 100% homology with the IS*900* insertion sequence for MAP (GenBank: S74401).

## Discussion

Using several different diagnostic techniques, we were able to identify and isolate MAP from nine individuals from three different ungulate species under human care in a zoo in Mexico.

Despite the cost and time required for bacterial cultures, this technique is considered the “gold standard test,” producing 100% specificity, with sensitivity varying depending on the sample and the growth medium used [10]. In this research, we obtained more positive fecal samples using bacterial cultures (12.5%) than with IS*900* PCR (3.5%). These results are similar to those of other studies, in which conventional PCR (cPCR) produced a lower sensitivity (30-50%) than bacterial cultures (70%) in the clinical phase of the disease [9,26]. However, more cases were detected in camelids when using PCR than with cultures, possibly because of contamination of the growth media used [27]. Recently, real-time quantitative PCR (qPCR) has been shown to have greater sensitivity than cPCR, even showing sensitivity equal to or greater than that of bacterial cultures [28,29]. Therefore, the use of qPCR is recommended, together with the optimization of methods for DNA extraction from feces, improving the sensitivity of MAP detection [30].

The nine MAP isolates obtained from the three ungulate species using type-specific PCR came from Type C. Type C MAP is known to have a wide range of hosts in both domestic and wild ruminants compared to other types, such as Type S, which has mainly been described in sheep and goats [31]. Genotype typification studies have detected Type C and its different subtypes in both red deer (*Cervus elaphus*) and scimitar-horned oryx [16,32]. These results agree with those in reports from Mexico, where Type C MAP has been detected in domestic bovine and caprine populations [20,23,33]. However, it is necessary to perform more extensive studies to understand the genetic diversity of MAP present in wild ruminants under human care in zoological collections, as well as in domestic ruminants in Mexico.

The finding of a positive IS*900* PCR result from the intestinal mucosa of a scimitar-horned oryx calf (identified as O9) suggests a possible intrauterine or transmammary transmission of MAP, given that this calf’s mother (O6) died a month after giving birth. Necropsy results from the mother showed MAP-related lesions, as well as positive bacterial isolation from the intestinal mucosa and feces, although the fecal-oral transmission route described in other studies cannot be ruled out in this case [13,34].

The fact that five out of 10 scimitar-horned oryx individuals produced positive isolates and positive IS*900* PCR tests from the intestinal mucosa indicates significant MAP infectiousness, as well as the susceptibility of this species. Other factors such as stress can contribute to PTB development in wild ruminants, similarly as reported for domestic species [26].

Fecal MAP isolation from a giraffe and two wildebeests showing no clinical signs indicated MAP exposure and probable infection. However, the infection could not be demonstrated by the observation of granulomatous intestinal lesions or intestinal MAP detection using culture or PCR tests [12,26]. It is important to note that MAP isolation from giraffe and wildebeest feces was detected until week 50 of incubation. This finding could be because the animals were in the initial phase of infection and thus producing a low concentration of MAP in feces. For this reason, it is recommended to maintain bacterial cultures under incubation for longer periods of time before ruling out infection. Since different MAP types grow better in different media, it is also valuable to use different growth mediums to increase the probability of obtaining MAP isolates.

## Conclusion

PTB could be a risk to the health and welfare of wild ruminants under human care, particularly for animals in conservation programs. The results obtained in this study show the importance of non-invasive individual fecal sample collections repeated over a period of time, the use of different diagnostic tests, and postmortem studies on animals deceased in zoos to understand the MAP status of wild ruminants under human care. This is the first study that has achieved MAP isolation and identification in wild ruminants in Mexico.

## Authors’ Contributions

ALH: Collected fecal samples, performed laboratory work, and wrote the manuscript. GC: Directed and supervised the project. EM: Supervised and provided support for laboratory and project work. LEA: Supported project work and obtained samples for histopathological studies. MTD: Supported project work and obtained samples for histopathological studies. All authors have read and approved the final manuscript.
